# Supplementary material for: Applying K-Means Cluster Analysis to Urinary Biomarkers in Interstitial Cystitis/Bladder Pain Syndrome: A New Perspective on Disease Classification
Source: Int J Mol Sci. 2025 Apr 14;26(8):3712. doi: 10.3390/ijms26083712 (PMC12028259; doi:10.3390/ijms26083712)
Supplement: Supplementary file 1 [file ijms-26-03712-s001.zip › ijms-3561492-supplementary.pdf]

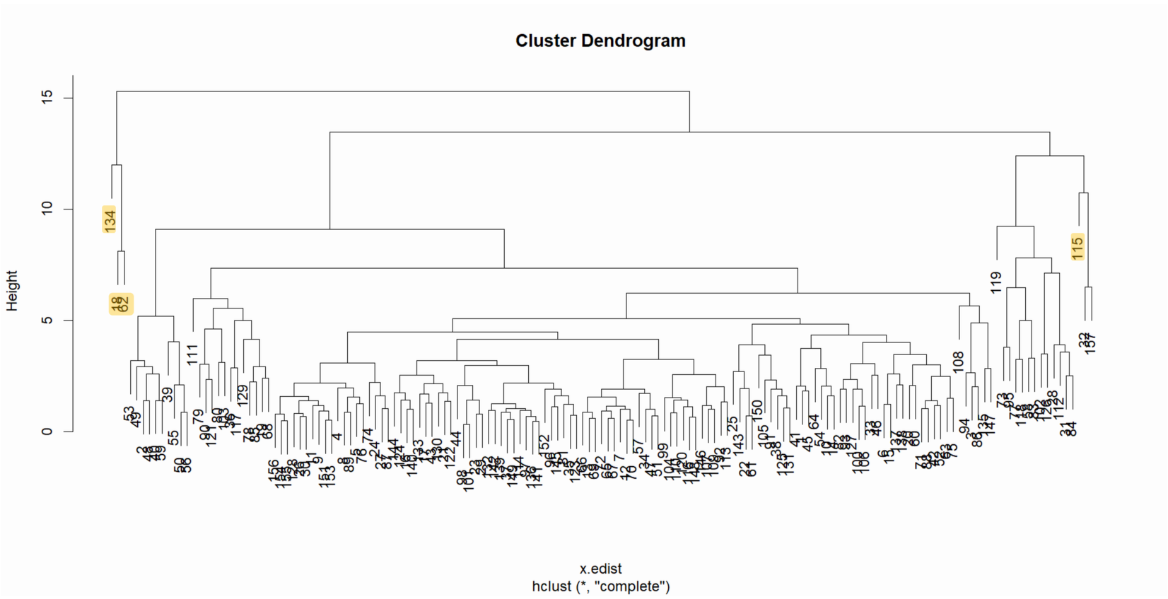

Supplement Figure S1. The hierarchical clustering of urinary biomarker profiles. It supported the inference that Cluster 3 represents outliers.

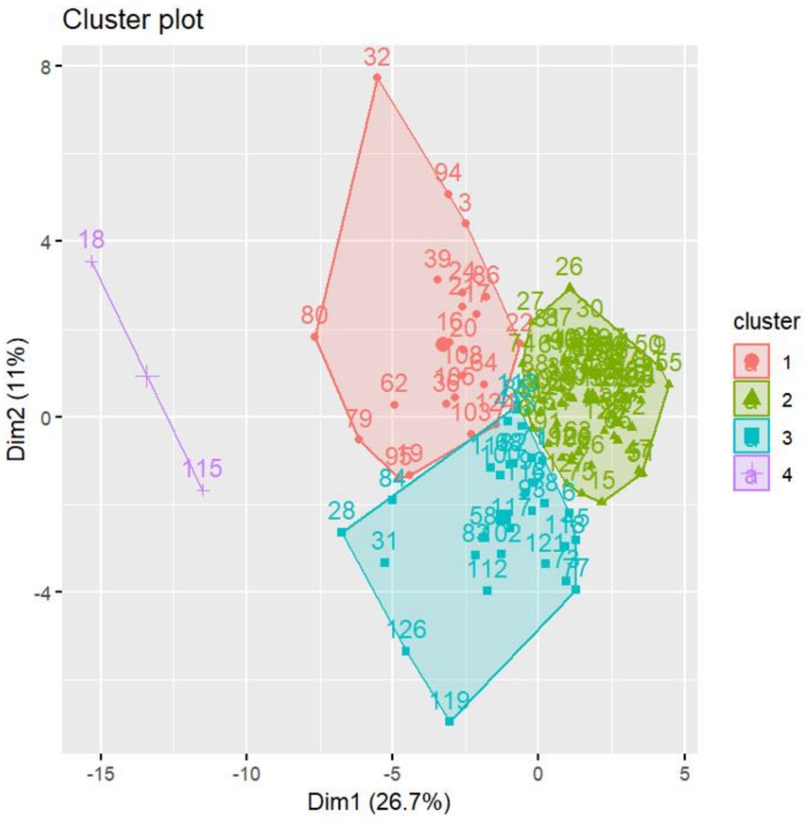

Supplement Figure S2. Cluster plot generated by K-means clustering of confirmed IC/ BPS patients, showing 4 clusters with sizes of 22, 73, 30, and 2, respectively.
